# Fosfomycin Resistance in Escherichia coli: Mechanisms, Trends, and Clinical Challenges

**DOI:** 10.7759/cureus.111969

**Published:** 2026-07-02

**Authors:** Gaurav N Patkar, Priyanka M Mane, Satish R Patil

**Affiliations:** 1 Department of Microbiology, Krishna Institute of Medical Sciences, Krishna Vishwa Vidyapeeth (Deemed to be University), Karad, IND

**Keywords:** antimicrobial stewardship, biofilm, esbl, fosa3, fosfomycin susceptibility, horizontal gene transfer, mdr e. coli, mura, upec, uti

## Abstract

Fosfomycin has reemerged as an important therapeutic option against multidrug-resistant (MDR) *Escherichia coli*, particularly extended-spectrum β-lactamase (ESBL)-producing strains causing UTIs. The rising incidence of carbapenem-resistant and ESBL-producing *E. coli *has reduced the number of therapeutic alternatives available and rekindled interest in fosfomycin as a successful therapy alternative, particularly for UTIs. Fosfomycin is a significant therapeutic drug in contemporary clinical practice due to its distinct mode of action, advantageous pharmacokinetic characteristics, oral availability, and high urine concentrations. However, isolates of *E. coli* have become resistant globally due to the widespread usage of fosfomycin. Resistance mechanisms include reduced drug absorption due to transport system mutations, changes to the *MurA* target enzyme, enzymatic inactivation due to *fos *genes, biofilm formation, and plasmid-mediated horizontal gene transfer. A significant obstacle to infection control and antimicrobial stewardship is the spread of resistance factors like *fosA3 *among MDR strains. The current knowledge on fosfomycin resistance in *E. coli*, including mechanisms of resistance, molecular genetics, epidemiological trends, laboratory detection techniques, therapeutic uses, and related clinical difficulties, is summarized in this study. In order to maintain the clinical usefulness of fosfomycin against resistant bacterial infections, the review also emphasizes the significance of antimicrobial stewardship, surveillance initiatives, and future research approaches. This narrative review summarizes literature published between 2010 and 2025 on resistance mechanisms, epidemiology, laboratory detection, therapeutic applications, and clinical challenges associated with fosfomycin-resistant *E. coli*.

## Introduction and background

Antimicrobial resistance (AMR), which increases morbidity and mortality, prolongs hospital stays, and raises healthcare costs, has become a significant global public health issue. The quick development of multidrug-resistant (MDR) Gram-negative bacteria has hampered the treatment of infectious diseases and drastically decreased the efficacy of widely used antibacterial medicines. The rising incidence of AMR in India has been mostly caused by improper antibiotic use, over-the-counter antimicrobial availability, and poor infection control procedures [1]. One of the greatest human pathogens, *Escherichia coli*, is a primary cause of hospital-acquired UTIs, bloodstream infections, and newborn meningitis. Nearly 70-80% of community-acquired UTIs and a sizable percentage of nosocomial UTIs in India are caused by uropathogenic *E. coli *(UPEC). The emergence of carbapenem-resistant and extended-spectrum β-lactamase (ESBL)-producing *E. coli *is a result of a paucity of treatment options and an increase in treatment failures. *E. coli *bacteria have become a significant therapeutic issue [2].

Among the most prevalent are UTIs. Bacterial disorders are seen in clinical practice, affecting millions of individuals globally. UTIs are a major cause of hospitalizations and outpatient visits in India, especially for women, the elderly, diabetics, catheterized patients, and immunocompromised people. The use of alternative medicines against resistant isolates has become necessary due to the growing incidence of AMR among uropathogens, complicating empirical therapy [3].

Due to its exceptional efficacy against MDR Gram-negative bacteria, particularly ESBL-producing *E. coli*, fosfomycin, an ancient broad-spectrum bactericidal antibiotic, has attracted fresh clinical attention. The medication has a number of benefits, including oral availability, high urine concentration, moderate toxicity, and less cross-resistance with other antibiotics. It inhibits the formation of bacterial cell walls by targeting the *MurA *enzyme. Fosfomycin has reappeared as a significant treatment option for both simple and complex UTIs brought on by resistant organisms because of these characteristics [4].

Fosfomycin-resistant strains of *E. coli *have emerged in recent years due to the increased use of fosfomycin against MDR and ESBL-producing bacteria. Target site alteration, plasmid-mediated *fos *genes like *fosA3*, and decreased drug absorption due to transporter mutations are examples of resistance mechanisms. The future use of fosfomycin is seriously threatened by the spread of these resistance factors, especially in nations like India, where antibiotic abuse and resistance are prevalent [5].

This narrative review aims to summarize current evidence regarding the mechanisms of fosfomycin resistance, molecular epidemiology, laboratory detection, therapeutic applications, and clinical challenges associated with *E. coli*. Particular emphasis is placed on emerging resistance trends in India within the broader global context.

## Review

Methodology

This narrative review was conducted through a literature search of PubMed, Scopus, Web of Science, and Google Scholar databases. Articles published between 2010 and 2025 were searched using the keywords “fosfomycin resistance”, “Escherichia coli”, “fosA3”, “multidrug-resistant E. coli”, “urinary tract infection”, “antimicrobial resistance”, and “fosfomycin susceptibility”. Relevant original research articles, review articles, surveillance studies, and clinical guidelines were included.

Overview of fosfomycin

Fosfomycin is a low-molecular-weight antibacterial derived from phosphonic acid that was first obtained from* Streptomyces fradiae *and related species. Several Gram-positive and Gram-negative bacteria, including MDR Enterobacterales, are susceptible to its broad-spectrum bactericidal action. Fosfomycin has a distinct antibacterial profile and less cross-resistance with other antimicrobial classes because it acts at an earlier stage of bacterial cell wall formation than glycopeptides and β-lactams [6].

The enzyme UDP-N-acetylglucosamine *MurA*, or enolpyruvyl transferase, is irreversibly inhibited by fosfomycin, which prevents the combination of N-acetylmuramic acid, a necessary precursor of peptidoglycan synthesis. Glycerophosphate transporter (GlpT) and glucose-6-phosphate transporter (UhpT) are two examples of systems for active transportation that allow the medication to enter bacterial cells. Fosfomycin is nevertheless effective against many MDR and ESBL-producing *E. coli* isolates that are resistant to aminoglycosides, cephalosporins, and fluoroquinolones because of this unique mechanism [7].

Fosfomycin comes in IV and oral forms. Because oral fosfomycin trometamol produces extremely high urine concentrations that continue for a long time following a single dosage, it is frequently used for simple UTIs. IV fosfomycin has become more important in treating severe systemic infections brought on by MDR *E. coli*, carbapenem-resistant Enterobacterales (CRE), and other challenging Gram-negative organisms. Carbapenems, aminoglycosides, colistin, or tigecycline are frequently used in combination therapy to increase antibacterial activity and reduce the emergence of resistance [8].

Fosfomycin’s advantageous pharmacokinetic and pharmacodynamic profile is one of its key features. Good tissue penetration, minimal protein binding, and unaltered renal excretion are all demonstrated by the medication. Furthermore, compared to many reserve antibiotics used against MDR infections, fosfomycin has a comparatively better safety record with fewer significant adverse effects. These characteristics have led to its growing therapeutic application globally, especially in areas where antibiotic resistance is more prevalent [9].

The extensive clinical usage of fosfomycin has sparked worries about the development of resistance during treatment, despite these benefits. Chromosome mutations that impact transport systems or the acquisition of plasmid-mediated *fos *genes can cause resistance to arise quickly. The possible threat to the long-term efficacy of fosfomycin is highlighted by the growing global identification of *E. coli* strains that possess *fosA3*. Therefore, to maintain this antibiotic’s therapeutic efficacy, sensible antibiotic use, precise susceptibility testing, and ongoing resistance monitoring are crucial [10].

*E. coli* as a major pathogen

*E. coli *is a facultative anaerobic, Gram-negative bacillus that belongs to the Enterobacteriaceae family and is a normal component of both human and animal gut flora. Pathogenic strains have many virulence characteristics that allow them to induce intestinal and extraintestinal infections, despite the fact that the majority of strains are benign commensals. Among the most common bacterial pathogens throughout the world that cause illnesses both in hospitals and within the community is *E. coli* [11].

About 70-90% of simple UTIs are caused by UPEC in community cases of UTIs. Virulence elements that aid colonization and persistence inside the urinary tract, including adhesins, fimbriae, toxins, iron acquisition systems, capsules, and biofilm development, are linked to the pathogenicity of UPEC [12].

*E. coli* is linked to bloodstream infections, intra-abdominal infections, pneumonia, newborn meningitis, and catheter-associated infections, in addition to UTIs. The growing prevalence of carbapenem-resistant, ESBL-producing MDR *E. coli *has become a significant public health issue because of the lack of effective treatments and the increase in treatment failures [13].

The global dissemination of high-risk clones such as ST131 has significantly contributed to the spread of AMR, including resistance to fosfomycin.

Mechanisms and molecular genetics of fosfomycin resistance

Fosfomycin resistance in *E. coli* is caused by a number of chromosomal and plasmid-mediated processes that lessen the drug’s antibacterial efficacy. Resistant isolates have emerged globally as a result of the growing clinical usage of fosfomycin against germs that produce ESBL and are MDR [7].

Reduced Drug Uptake

Reduced intracellular uptake of fosfomycin as a result of mutations in the membrane transport systems GlpT and UhpT, which are in charge of the drug’s active transport into bacterial cells, is one of the most prevalent resistance mechanisms. Changes in these transporters dramatically lower susceptibility and minimize fosfomycin entrance [10].

Target Modification

Another important mechanism involves the modification of the target enzyme *MurA*. Fosfomycin irreversibly inhibits *MurA*, an enzyme required for the first step of peptidoglycan synthesis. Mutations in the *MurA *gene may reduce fosfomycin binding and thereby decrease antibacterial activity [10].

Enzymatic Inactivation

Another important resistance mechanism is enzymatic inactivation mediated by enzymes encoded by *fos *genes, which are frequently carried on plasmids. *Fos *genes, including *fosA3*, *fosB*, and *fosC2*, encode enzymes that catalyze the addition of glutathione or other substances to the antibiotic molecule, rendering fosfomycin inactive. Among these, *fosA3 *is commonly associated with *E. coli *that generate ESBL and promote the rapid horizontal gene transfer of resistance [14,15].

Mobile Genetic Elements and Horizontal Gene Transfer

Biofilm formation contributes to persistence and reduced antibiotic penetration. In addition, mobile genetic elements such as plasmids, transposons, and integrons facilitate the dissemination of fosfomycin resistance genes among Enterobacterales. These elements promote horizontal gene transfer and contribute substantially to the global spread of AMR [16] (Table 1).

**Table 1:** Mechanisms of fosfomycin resistance

Mechanism	Gene	Effect	Transferable
Reduced uptake	GlpT, UhpT	Reduced intracellular concentration	No
Target modification	MurA	Reduced drug binding	No
Enzymatic inactivation	*fosA*, *fosA3*	Drug degradation	Yes
Biofilm formation	Multiple	Reduced penetration	No
Horizontal transfer	Plasmids	Spread of resistance	Yes

Laboratory detection of fosfomycin resistance

Fosfomycin resistance in *E. coli* must be accurately detected in the lab in order to monitor resistant organisms and administer the proper antibiotics. Fosfomycin susceptibility testing is difficult because the results can change based on the growth conditions and testing technique. Reliable detection, therefore, requires standardized laboratory methods and interpretation based on guidelines [17,18].

For urinary *E. coli *isolates, the Clinical & Laboratory Standards Institute (CLSI) defines fosfomycin susceptibility as a minimum inhibitory concentration (MIC) of ≤64 µg/mL, intermediate susceptibility as 128 µg/mL, and resistance as ≥256 µg/mL. Due to its labor-intensive nature, most clinical microbiology labs do not routinely employ this approach, despite the fact that it produces reliable MIC values [19].

Due to its ease of use and affordability, disk diffusion testing is frequently utilized in regular laboratories. Glucose-6-phosphate, which increases the drug’s active transport into bacterial cells and improves test accuracy, must be present in fosfomycin disks used for susceptibility testing. To prevent reporting of misleading susceptibility or resistance, interpretation should be done in accordance with CLSI or European Committee on Antimicrobial Susceptibility Testing breakpoints [20]. A recognized limitation of fosfomycin susceptibility testing is occasional discrepancy between disk diffusion and MIC-based methods, particularly among isolates exhibiting heteroresistance.

Fosfomycin testing can also be done using automated susceptibility systems and broth microdilution, albeit there may be differences with agar dilution techniques. Interpreting susceptibility results can be made more difficult by heteroresistance and the formation of resistant subpopulations during testing [19].

Molecular methods such as polymerase chain reaction, whole genome sequencing (WGS), and multiplex molecular tests are quickly identifying fosfomycin resistance genes, such as *fosA*, *fosA3*, and other plasmid-mediated determinants. These techniques are helpful for epidemiological studies and tracking the spread of resistance among isolates of MDR *E. coli *[15]. The presence of *fos *genes does not necessarily indicate phenotypic resistance because gene expression may vary among isolates.

Accurate diagnosis and monitoring of fosfomycin resistance in clinical settings require ongoing quality control, adherence to established protocols, and integration of molecular diagnostics [18].

Epidemiology of fosfomycin resistance

Global Trends

The growing clinical usage of fosfomycin for MDR infections has significantly altered the global epidemiology of fosfomycin resistance in *E. coli *over the past 10 years. Fosfomycin resistance rates are still very low when compared to many other antibiotics, but an increasing number of resistant isolates have been reported worldwide, particularly among carbapenem-resistant and ESBL-producing *E. coli *[18].

The use of antibiotics, infection control strategies, and antimicrobial stewardship regulations all affect resistance rates geographically. Higher rates of resistance have been noted in Asian nations, such as China and India, where widespread use of antibiotics and the transmission of resistance genes via plasmids play a major role in the proliferation of resistant strains. Fosfomycin resistance has spread more quickly throughout the world among Enterobacterales due to the discovery of transferable genes like *fosA3 *[16].

Fosfomycin maintains strong in vitro efficacy against urinary *E. coli *isolates, particularly in simple UTIs, according to a number of surveillance studies. On the other hand, hospital-acquired isolates, recurrent UTIs, catheter-associated infections, and ESBL-producing pathogens are increasingly reported to have resistance. The proliferation of resistance determinants throughout healthcare settings has been exacerbated by the increasing frequency of MDR clones like ST131 [14].

The global epidemiology of fosfomycin resistance in *E. coli *has evolved considerably over the last decade due to increased clinical use of fosfomycin for the treatment of MDR infections. Although resistance rates remain lower than those reported for fluoroquinolones and third-generation cephalosporins, resistant isolates are increasingly being reported worldwide, particularly among ESBL-producing and carbapenem-resistant strains [18].

Several surveillance studies have demonstrated that fosfomycin retains excellent in vitro activity against urinary *E. coli *isolates, with susceptibility rates often exceeding 90-95% in many European and North American settings. However, resistance rates ranging from 5% to 20% have been reported among ESBL-producing isolates and hospital-acquired strains in several Asian countries [16,18].

The emergence and dissemination of plasmid-mediated resistance determinants such as *fosA3*, together with the spread of high-risk clones, including ST131, have contributed substantially to the global burden of fosfomycin resistance [14,16].

Indian Scenario

Fosfomycin resistance among isolates has become a serious concern in India due to the increasing prevalence of MDR and ESBL-producing strains of *E. coli*. Several Indian studies have demonstrated high susceptibility rates of *E. coli *to fosfomycin despite increasing AMR. Gupta et al. reported susceptibility rates exceeding 90% among ESBL-producing UPEC isolates, supporting the continued utility of fosfomycin in the treatment of resistant UTIs [4].

Fosfomycin maintains good in vitro action against UPEC, including isolates that produce ESBL, according to a number of Indian studies. Resistance rates vary across different regions and healthcare settings. While community-acquired urinary isolates generally demonstrate high susceptibility, comparatively higher resistance rates have been reported among hospital-acquired isolates, recurrent UTIs, and ESBL-producing *E. coli*. Soni et al. also demonstrated preserved fosfomycin activity against ESBL-producing Enterobacterales in a tertiary care setting, although resistant isolates were identified [21].

The emergence of plasmid-mediated resistance determinants such as *fosA3 *is of particular concern because these genes can spread rapidly through horizontal gene transfer. Several studies from Asia have reported increasing detection of *fosA3*-carrying plasmids among ESBL-producing *E. coli*, highlighting the need for continuous molecular surveillance [16,17].

Inadequate antimicrobial stewardship programs, self-medication, over-the-counter drug availability, and improper antibiotic prescribing practices all play a major role in the spread of resistance in India. Underreporting of resistance isolates may also be caused by the lack of routine fosfomycin susceptibility testing in many microbiology labs [2].

The available evidence suggests that fosfomycin resistance in India remains lower than resistance to many other commonly used antimicrobials; however, increasing resistance among ESBL-producing and MDR *E. coli* isolates warrants continued surveillance. Strengthening infection-control measures, improving laboratory detection methods, expanding routine susceptibility testing, and implementing antimicrobial stewardship programs are essential to preserve the effectiveness of fosfomycin in India [22,23].

Clinical challenges and therapeutic role of fosfomycin

The increasing prevalence of fosfomycin-resistant *E. coli *presents significant therapeutic challenges, particularly in the management of complicated UTIs and healthcare-associated infections. Although fosfomycin remains an effective treatment option for many resistant isolates, inappropriate or prolonged use may contribute to treatment failure and selection of resistant subpopulations during therapy [5].

Because susceptibility results can differ depending on the testing methods utilized, it is still challenging to accurately detect fosfomycin resistance in laboratories. Heteroresistance among bacterial isolates, a lack of standardized testing facilities, and a lack of systematic susceptibility testing in many clinical laboratories can all confound interpretation and cause treatment decisions to be delayed [19].

The quick spread of plasmid-mediated resistance genes, like *fosA3*, which promote horizontal resistance transmission across Enterobacterales, is another significant obstacle. The necessity for efficient antimicrobial stewardship and ongoing surveillance programs is highlighted by the growing resistance among hospital-acquired isolates, recurrent UTIs, and catheter-associated infections, which further restricts the oral treatment options [16].

Because of its broad-spectrum action against MDR *E. coli *and other Gram-negative bacteria, fosfomycin has resurfaced as a significant therapeutic agent. Fosfomycin is frequently used to treat both simple and complex UTIs, especially those brought on by ESBL-producing *E. coli*, because of its distinct mode of action and low cross-resistance with other antibiotic classes [18].

Because oral fosfomycin trometamol produces high urine concentrations and has good clinical performance with single-dose administration, it is frequently advised as a first-line treatment for uncomplicated cystitis. In order to improve treatment results and stop the establishment of resistance, IV fosfomycin is being used more frequently in cases of severe systemic infections, such as bacteremia, complex UTIs, and infections brought on by CRE. Oral fosfomycin trometamol is primarily used for uncomplicated UTIs, whereas IV fosfomycin disodium is reserved for severe systemic infections caused by MDR organisms [5].

Fosfomycin is especially helpful for patients with few oral therapy alternatives because of its advantageous pharmacokinetic characteristics, which include minimal toxicity, good tissue penetration, and excellent urinary excretion. The necessity for susceptibility-guided medication and antimicrobial stewardship is highlighted by the emergence of resistance during monotherapy and the rise in plasmid-mediated resistance [8].

Combination therapy involving fosfomycin with carbapenems, colistin, aminoglycosides, or tigecycline has demonstrated synergistic activity against selected MDR Gram-negative pathogens, including CRE. Such combinations may improve treatment outcomes and reduce the likelihood of resistance development; however, additional clinical studies are required to establish optimal therapeutic regimens [5,8].

Antimicrobial stewardship and prevention

In order to prevent fosfomycin-resistant strains of *E. coli *from emerging and spreading, antimicrobial stewardship is essential. To maintain fosfomycin’s efficacy against isolates that produce ESBL and MDR, rational use of antibiotics, avoidance of needless empirical therapy, and susceptibility-guided treatment are crucial. Hospital antibiotic policies and ongoing surveillance initiatives can be put into place to track resistance patterns and maximize the use of antibiotics [23].

In India, surveillance initiatives such as the Indian Council of Medical Research Antimicrobial Resistance Surveillance and Research Network play an important role in monitoring resistance trends, generating national AMR data, and guiding antimicrobial stewardship strategies.

Reducing the spread of resistant *E. coli* strains in hospital settings requires strict infection prevention and control measures, such as hand cleanliness, catheter care, ambient sanitation, and isolation precautions. Additionally, limiting the availability of over-the-counter antibiotics and raising public awareness of proper antibiotic use are essential to reducing antimicrobial overuse and halting the spread of resistance [2].

Future perspectives

Fosfomycin-resistant *E. coli *infections are on the rise, which emphasizes the necessity of ongoing research and the creation of practical solutions to maintain fosfomycin’s clinical usefulness. The molecular epidemiology of resistance genes, particularly plasmid-mediated determinants like *fosA3*, and their function in the spread of MDR strains should be the main focus of future research. In both hospital and community settings, WGS and advanced genomic surveillance may aid in the early identification and tracking of resistant clones [16].

Accurate diagnosis of fosfomycin resistance and prompt therapeutic decision-making depend on the development of quick molecular diagnostic approaches and uniform susceptibility testing procedures. Affordable molecular assays for rapid detection of *fosA3 *and other transferable resistance determinants remain limited in many resource-constrained settings and should be prioritized to improve early detection and surveillance of fosfomycin resistance. Additionally, investigating fosfomycin-based combination therapies against carbapenem-resistant and ESBL-producing* E. coli *may enhance treatment results and lessen the emergence of resistance during therapy [7].

Future goals should include bolstering antimicrobial stewardship initiatives, encouraging sensible antibiotic usage, and carrying out multicentric surveillance investigations, especially in emerging nations like India. To maximize fosfomycin’s therapeutic function against resistant Gram-negative organisms, more clinical trials assessing the drug’s effectiveness and safety in severe systemic infections are required [5].

Limitations

This review has several limitations. As a narrative review, it does not employ systematic review methodology and may therefore be subject to selection bias. Quantitative meta-analysis was not performed, and available Indian epidemiological data remain limited and heterogeneous across studies.

## Conclusions

Fosfomycin’s comeback has been a crucial lifeline in today’s therapeutic environment, providing an efficient broad-spectrum bactericidal substitute against *E. coli *that produces ESBL and is MDR. It is particularly useful for treating complex UTIs due to its distinct method of action, excellent safety profile, and high urine concentrations. However, the widespread emergence of resistance poses a growing threat to this agent’s therapeutic duration. The development of highly transmissible, plasmid-mediated determinants like *fosA3* by horizontal gene transfer and chromosomal mutations that change target enzymes like *MurA *or interfere with GlpT and UhpT membrane transport systems are the two main causes of fosfomycin resistance in *E. coli*. High baseline AMR rates, improper empirical usage, and systematic inadequacies in routine laboratory testing and reporting exacerbate this problem in places like India. Future multicenter surveillance studies, improved molecular diagnostics, and strengthened antimicrobial stewardship programs are essential to preserve the effectiveness of fosfomycin against MDR *E. coli *infections.
